# Esophageal magnetic compression anastomosis for esophageal atresia repair: when not to use magnets—our experience

**DOI:** 10.1007/s00464-025-12033-4

**Published:** 2025-08-07

**Authors:** Anne-Sophie Bernadette Holler, Jacqueline C. Kading, Michael R. Harrison, Oliver J. Muensterer

**Affiliations:** 1 Department of Pediatric Surgery, Dr. von Hauner Children’s Hospital, University Hospital, LMU Munich, Lindwurmstr. 4, 80337 Munich, Germany; 2 Neonatology, Department of Pediatrics, University of California San Francisco, San Francisco, CA USA; 3 Department of Pediatric Surgery, University of California San Francisco, San Francisco, CA USA

**Keywords:** Esophageal atresia, Magnetic compression, Pediatric surgery, Endoscopy, Minimally invasive, Thoracoscopy

## Abstract

**Background:**

Esophageal magnetic compression anastomosis (EMCA) represents an innovative minimally invasive approach for establishing esophageal continuity for patients with esophageal atresia, especially when comorbidities increase the risk of a thoracoscopic or open operation. Over the last 5 years, we have demonstrated favorable outcomes in our patients, but have also encountered cases in which EMCA was not successful. This report is an in-depth analysis of the failed EMCA attempts.

**Methods:**

A retrospective chart review of all patients who were scheduled for EMCA was performed. All patients who failed magnet placement and underwent conventional repair instead were identified and presented as case series. Patient characteristics, intra- and postoperative data, reasons for failure, and follow-up data were analyzed.

**Results:**

From 2021 through 2024, EMCA was attempted in a total of 15 patients by our team on an intention-to-treat basis under compassionate care ethics. Placement of the magnets failed in 3 patients (20%). Every case showed specific characteristics that prohibited magnet coupling. In case 1, metal clips that had been placed during a prior lengthening procedure precluded mating of the magnets. In case 2, an atretic lower pouch was present and therefore the magnet could not be advanced toward the proximal end of the distal pouch. In the third case, an atypical bronchus was interposed between the two esophageal pouches, resulting in distance and tissue between the upper and lower pouch.

**Conclusion:**

EMCA is a minimally invasive approach for endoscopic esophageal anastomosis creation in complex cases of esophageal atresia, or patients with comorbidities that confer a very high surgical risk. Careful patient selection and preparation is crucial to maximize the chance of success. Based on our current experience and case series, we propose certain anatomic variants and pre-existing factors as contraindications for EMCA.

Esophageal magnetic compression anastomosis (EMCA) was first described in 2009 by Zaritzky et al., and since then has undergone multiple changes and adoptions. The method offers an endoscopic, minimally invasive approach to achieve esophageal anastomosis [1, 2]. In the beginning, magnets were used for both approximation, elongation, and subsequent esophageal anastomosis. Unfortunately, long-term follow-up of these patients revealed a 100% stricture rate with the need of a high number of dilatations (average 9.8 dilatations per patient). Therefore, the approach was mostly abandoned [3].

Refinements in magnetic design and configuration, as well as the evolution of the procedure to include prior approximation of the pouches before placement of the magnets lead to a revival of the technique by our group. After thorough preclinical testing, successful first in-human experiences were reported [4, 5]. The outcomes of the initial patients demonstrated safety and feasibility with shorter exposure to general anesthesia [5] and lower post-anastomotic dilatation requirements [6]. The key principle of the novel technique is the application only for creation of the anastomosis and explicitly not for approximation and lengthening. Therefore, in most cases, approximation of the esophageal pouches is performed before EMCA [4, 5].

In our series, we noticed that magnet placement was not possible in some patients. This report describes the cases in which EMCA was unsuccessful and analyzes possible contributing factors that could represent contraindications for EMCA in the future.

## Patients and methods

### Study cohort

This is a case series of patients in whom EMCA was not successful. A retrospective chart review of all patients who were scheduled for esophageal magnetic compression anastomosis was performed. All patients who failed magnet placement and underwent conventional repair instead were identified and reasons for unsuccessful magnetic intervention were analyzed. Patients who had successful magnet placement and anastomosis were excluded.

### Variables

Patient characteristics were collected retrospectively. Gender, birth weight, gestational age, type of atresia, comorbidities, and interventions prior to EMCA were retrieved from the electronic case records. Intraoperative and postoperative data, reason for failure, and follow-up data were analyzed. Descriptive statistics were performed and key points of EMCA failure were postulated.

### Device

In all patients, EMCA was attempted using the Connect-EA device (Myka Labs, San Francisco, CA) [4]. This novel device exhibits a special shape and tissue compression profile. It consists of two 8-mm-diameter magnetic anchors with a neodymium–iron–boron magnetic core which are encapsulated by biocompatible encasing, including a gold surface with parylene coating.

### Operative technique

The procedure was performed under general anesthesia. First, the gap length was assessed by radiographic gap measurement. Therefore, a 5-mm endoscope was introduced through the gastrostomy site and directed to the end of the lower esophageal pouch. The upper esophageal pouch was visualized on X-ray through a Replogle tube or a second endoscope. When minimal distance (below 10 mm) was confirmed, the first magnet was inserted in the upper pouch with an endoscopic grasper. Consequently, the second magnet was placed in the lower pouch via the gastrostomy and the gastroesophageal junction in the same manner. If needed, the gastrostomy site was dilated to 7 mm using Hegar dilators before magnet insertion. Both magnets were pushed together slightly until they attracted each other by magnetic force. All steps were monitored under intraoperative fluoroscopy. When the magnets successfully mated, all instruments were retrieved and a Replogle tube was placed in the upper pouch to prevent aspiration. If no mating occurred, the magnets were retrieved and the procedure was concluded.

## Results

From 2016 through 2024, a total of 15 patients were deemed candidates for EMCA. In 3 of these cases, the mating of the magnets was unsuccessful, so that the procedure was aborted and a hand-sewn anastomosis was performed. Details of the 3 patients are narratively described and summarized (Tables 1 and 2).

**Table 1:** Demographic data

Patient	1	2	3
Gender	Male	Male	Female
Gestational age (weeks)	38 + 5	36 + 1	37 + 3
Birth weight (g)	2900	2000	2530
EA type	EA with proximal tracheoesophageal fistula	EA with distal tracheoesophageal fistula	EA with distal tracheoesophageal fistula
Associated anomalies			
*Vertebral*	cleft vertebrae (thoracic vertebra)	13 ribs	
*Anorectal*		Anorectal malformation with rectovesical fistula	
*Cardiac*	Atrial septal defect	Ventricular septal defect, atrial septal defect	Atrial septal defect
*Renal* *Limb*		Single kidney, vesicoureteral reflux	
Procedures prior to intervention	- Open gastrostomy, gastric perforation and peritonitis - Laparotomy and lavage - Inguinal exploration and inguinal hernia closure + right orchidopexy - Thoracoscopy and closure of upper pouch fistula, approximation of both esophageal ends - 2 × thoracoscopy and approximation of both esophageal ends	- Thoracotomy with closure of the tracheoesophageal fistula and traction of the lower pouch on the thoracic wall, colostomy formation, and placement of a gastrostomy - Recurrent fistula, open fistula closure and traction of both pouches - Thoracotomy and repeat approximation	- Thoracotomy with closure of tracheoesophageal fistula, approximation of both esophageal ends, gastrostomy placement

**Table 2:** Procedural data

Patient	1	2	3
EMCA (number of attempts)	2	4	2
Reason for failure	Metal clips Distance between pouches	Narrow lower pouch, magnet could not be advanced through the most distal lower portion of the esophagus	Atypical bronchus
Alternative approach	Thoracotomy and end-to-end anastomosis	Thoracotomy and end-to-end anastomosis	Thoracoscopic end-to-end anastomosis

### Patient 1

**Fig. 1:**
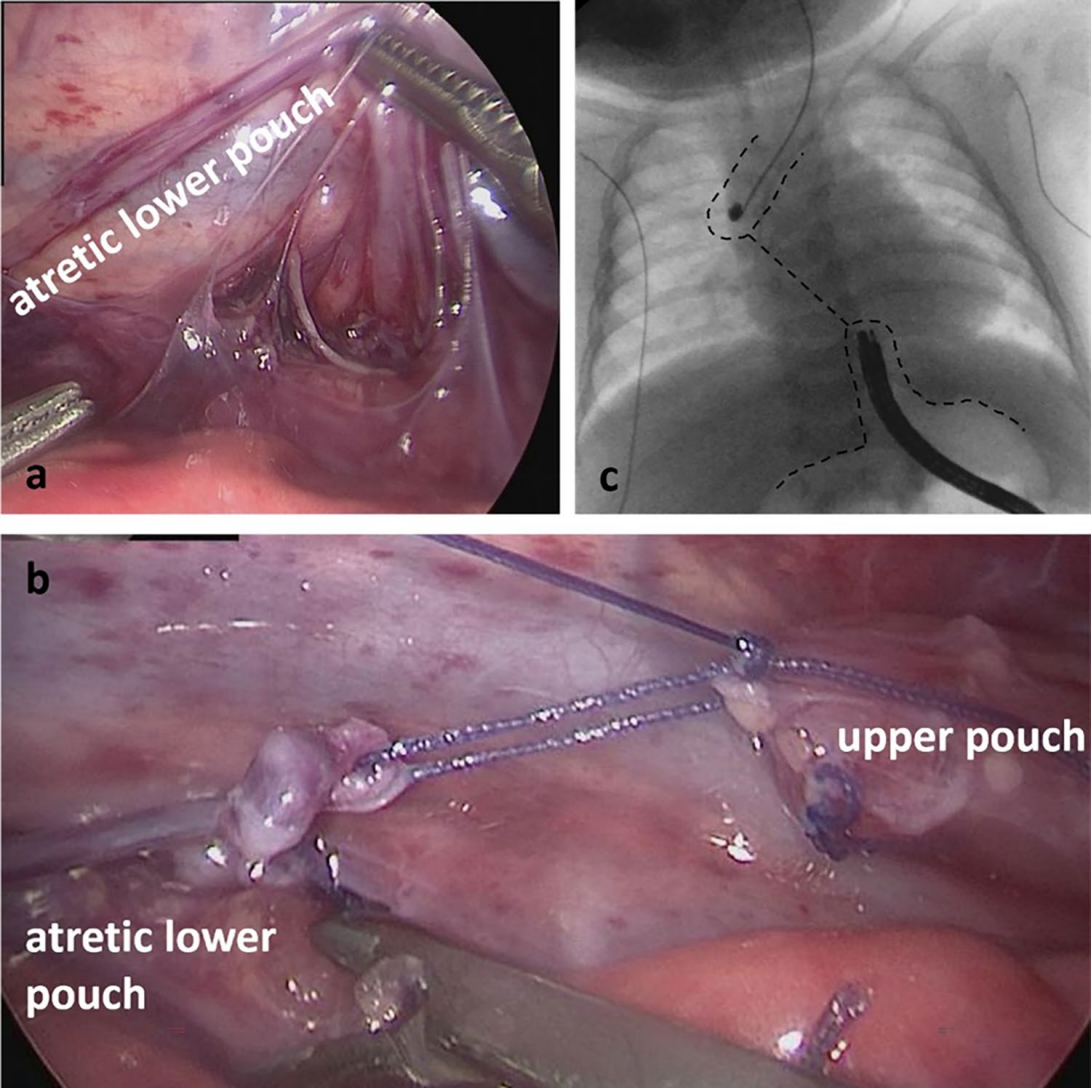
Patient 1: **a**, **b** Intraoperative view during first thoracoscopy, atretic lower pouch marked. Approximation of both esophageal pouches shown. **c** Gap-o-gram before 3rd traction procedure

Patient one was a term born male patient with long-gap EA with proximal tracheoesophageal fistula. He was born in an outside hospital where he had a gastrostomy placed and subsequently was referred to our hospital. Bronchoscopy and esophagoscopy were performed and an upper pouch fistula was detected. Subsequent thoracoscopy showed a very short distal esophagus with an atretic segment without a lumen (Fig. 1). The long atretic segment reached up to the same point where a distal tracheoesophageal fistula usually enters the trachea. Thoracoscopic approximation and proximal fistula closure was performed. Thoracoscopic approximation was repeated twice (at 6 and 12 weeks, respectively). After the last approximation, the two pouches could be brought close together. Technically, during the last approximation, two metal clips were placed at the end of the esophageal pouches to evenly distribute the tension of the traction sutures when performing the approximation. After the tension was thought to have subsided, endoscopic placement of the Connect-EA device was planned. Intraoperatively, mating of the two magnets could not be achieved. In a second attempt 3 weeks later, mating could still not be achieved, so that we proceeded with open thoracotomy and esophageal anastomosis. The postoperative course was complicated by postoperative chylothorax which resolved under medical treatment. A postoperative anastomotic stricture was treated with serial dilatations (11 dilatations, twice combined with steroid application) and stent placement. At last follow-up, the patient was on partial oral and gastral feeds through the gastrostomy.

### Patient 2

A twin boy was born at 36 weeks of gestation, a birth weight of 2000 g, and EA with distal tracheoesophageal fistula, anorectal malformation with rectovesical fistula, ventricular septal defect, atrial septal defect, and single kidney. On day 2 of life, a thoracotomy with closure of the tracheoesophageal fistula and internal traction on the lower pouch was performed, along with colostomy formation and placement of a gastrostomy. At 8 weeks of age, a recurrent fistula was detected and the boy underwent open fistula closure and internal traction of both pouches. At age 3 months (15 weeks) of age, an esophageal anastomosis was planned, but had to be abandoned due to too much tension on the pouches, so that an approximation procedure was performed instead in preparation of future anastomosis.

**Fig. 2:**
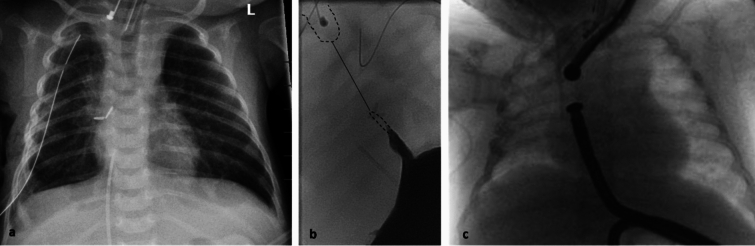
Patient 2: **a**, **b** Post- and intraoperative radiologic images after the first approximation procedure—long distance of about 4 vertebral bodies is clearly visible. **c** Intraoperative views of attempted EMCA—distance of about 1 vertebral body still present

At age 5 months, endoscopic placement of two magnets was attempted, but failed. There was still a gap of approximately one vertebral body, precluding the mating of the magnets (Fig. 2). A total of 4 EMCA attempts were made. Three interventions were carried out in context with other procedures – tracheostomy, laparoscopic-assisted anorectoplasty for anorectal malformation and colostomy closure. At the last attempt, a very narrow end of the lower pouch was detected, which was passable with a wire, but not with the 8-mm magnet.

Finally, at age 7 months, a thoracotomy and esophageal anastomosis was performed. The postoperative course was uneventful and an esophagogram on postoperative day 6 showed patency of the anastomosis with no leakage. Since then, the gastrostomy has been closed and the boy is on full oral feeds.

### Patient 3

**Fig. 3:**
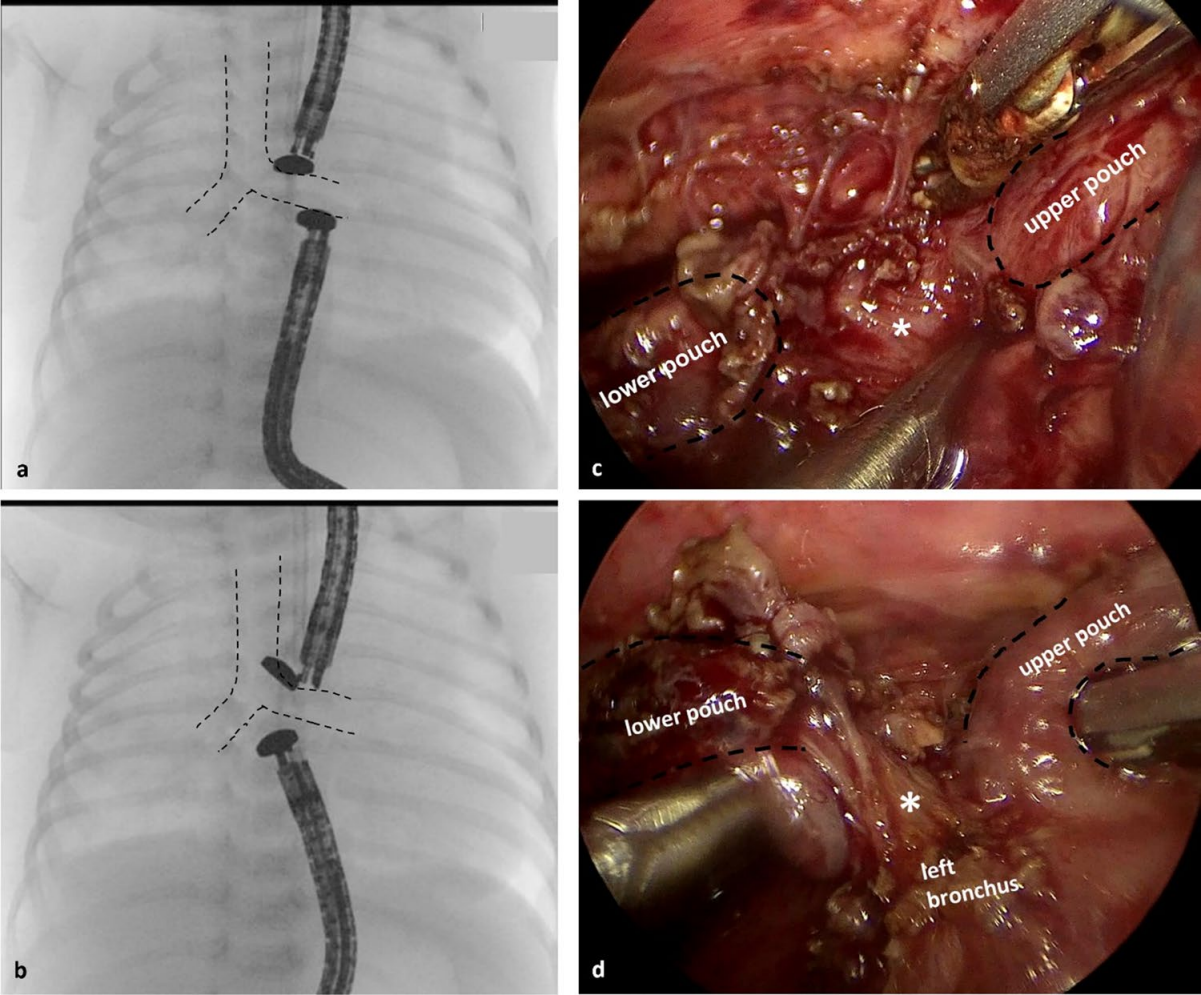
**a**, **b** Intraoperative fluorsocopy images of attempted magnet placement. **c**, **d** Thoracoscopic view. Interposition of the left mainstem bronchus and suture material (*) between the lower and upper pouch are clearly visible

A 2530 g girl was born at 37 weeks of gestation and EA with distal tracheoesophageal fistula at an outside hospital. Thoracotomy with closure of the fistula and approximation of both esophageal ends was performed on day 5 of life. Intraoperatively, presence of a long-gap esophageal atresia was determined and therefore, a primary anastomosis was not attempted. A gastrostomy was placed during the same anesthesia. For further treatment, the girl was referred to our tertiary center. The following course was complicated by chylothorax which resolved under medical treatment after 5 weeks. The girl underwent two attempts of unsuccessful magnet placement, both failing due to the inability to get the magnets mated. Although the distance of the two esophageal ends seemed very close (Fig. 3).

Following the last attempt, the girl underwent thoracoscopic esophageal anastomosis in the same anesthesia. During thoracoscopy, an atypical course of the left main bronchus was visible which was interposed between the two esophageal ends. For that reason, the two magnets could not be mated. Postoperatively, the girl showed an anastomotic leakage which resolved spontaneously after 14 days and oral feeding was started.

## Discussion

Magnets are an innovative, minimally invasive approach to create an anastomosis in EA patients who have comorbidities that place the child at risk of prolonged anesthesia. Esophageal magnetic compression anastomosis was first described by Zaritzky et al., who used the magnetic force to both approximate the ends and create the anastomosis in one procedure. This approach ultimately was abandoned due to severe recurrent anastomotic stenosis that needed a high number of dilatations, stenting, and resection with reanastomosis in some cases [1, 3].

Our own technique entails prior approximation of the pouches without anastomosis, waiting for the tension to subside, and then place magnets to perform a reproducible, secure, and watertight anastomosis. Also, the shape of the magnets we use is unique in that it combines a high-pressure central zone with lower compression pressures at the periphery, allowing for healing and mucosal bridging.

However, over the last years, and during the first 15 cases, we noticed that some procedures were unsuccessful. The aim of this study was therefore to share our experience and highlight the limits and pitfalls of EMCA. This series describes three cases in which esophageal magnetic compression anastomosis failed. All patients exhibited comorbidities and risk factors for long anesthesia duration and sutured esophageal anastomosis that made them suitable candidates for EMCA.

In the first case the lower pouch was extremely short and approximation was difficult. Furthermore, we used metal clips for approximation. We think having metal clips placed, prevents or at least complicates placement of the magnets. The second case had a very thin lower pouch, so that the magnet could not be forwarded to the end of the pouch and therefore mating was impossible. In the last case, anatomic anomalies precluded success of EMCA. In our opinion forceful mating of the magnets should be avoided as this will lead to an anastomosis under tension and worsens stricture formation. In these cases, an alternative approach should be considered.

All three cases met the criteria for long-gap EA. Many approaches for long-gap EA have been described in the literature so far. Among those are delayed repair, lengthening procedures, and gastric, colonic, or jejunal interpositions. ERNICA guidelines for the management of long-gap EA declare preservation of the native esophagus as primary goal before considering any replacement technique as *“no other conduit can replace its function in transporting food from the oral cavity to the stomach satisfactorily” *[9, 10]. A systematic review comparing gastric interposition and esophageal lengthening procedures described a 58% esophageal anastomotic stricture/stenosis rate and an 17% anastomotic leak rate for lengthening procedures and a 16% esophageal anastomotic stricture/stenosis rate and 12% anastomotic leak rate for gastric interpositions [7]. Reddy et al. reported a series of 10 patients with long-gap EA who were treated with thoracoscopic external traction suture elongation. In this series, 5/10 required dilatations and 3 had anastomotic leakage [8].

All these studies highlight the fact that management of long-gap EA is challenging and stricture formation and leakage are major and frequent complications. The main downside of EMCA is stricture formation, whereas so far, no anastomotic leakages occurred [4, 5].

The number of procedures and length of anesthesia exposure may influence long-term neurologic outcome of these patients. One downside of the traditional Foker process is that the children remain sedated and paralyzed in the ICU while on traction [11]. With serial internal approximation, long-term sedation is not necessary and the EMCA procedure itself, in our hands, had a median duration of 30 min (range: 20–54 Minutes) [5].

An algorithm to optimize the advantages of the device for EA repair was proposed by Lee et al. [5]. This included the recommendation to use only absorbable suture material for pouch approximation and to avoid metal clip application. Our experience showed that this recommendation should be extended by cautious application in patients with anatomic anomalies and extremely narrow pouch ends. In general, we place the upper magnet first, because it cannot dislocate. We then carefully insert the lower magnet with the patient in a 20° right-side up position to minimize the chance of transpyloric dislocation of the magnet in case it detaches from the endoscope.

From the experience with the reported 3 patients, we have learned that exact and diligent preparation of both pouches is paramount to maximize the success rate of EMCA. In the ideal situation, the surfaces of both pouches should be in direct juxtaposition, with small (4–0 or 5–0) resorbable traction sutures placed circumferentially around the mating surface. We usually place 3 or 4 sutures using a slip-knot technique and then slowly tighten the sutures sequentially several times to bring the ends of the pouches together. Any material between the pouches, such as clips or particularly non-resorbable suture material, must be avoided. Finally, exact knowledge of the anatomy of the pouches and the surrounding structures is necessary to prevent ineffective mating and complications.

In general, EMCA is applicable to all patients with esophageal atresia for the creation of esophageal anastomosis, after the esophageal pouches have been approximated and tracheoeosophageal fistulas have been closed. In our opinion, especially patients with long-gap EA, comorbidities that pose them at special anesthetic risks and after failed thoracotomy and anastomosis benefit the most. Due to the small magnet size (8 mm), there is no lower weight limit.

The limitation of our study is the low number of patients described in our series. However, this is the largest series of failed EMCA attempts reported, and we do believe that the lessons learned are worth noting to avoid complications in future EMCA procedures.

Another important limitation of this report is the potential for bias due to conflicts of interest. Two authors are involved in the development of the EMCA device used in this study. However, this series specifically highlights failed cases and procedural limitations, which we believe reflecting a commitment to transparent reporting and responsible device use.

## Conclusion

EMCA is a minimally invasive approach for esophageal anastomosis in selected complex cases of esophageal atresia. However, in our experience, several pitfalls should be avoided. Direct juxtaposition of the pouches is mandatory for successful later magnet placement. Interposed material such as clips, suture material, or adjacent tissue such as a bronchus must be avoided. Care must also be taken to remove atretic segments of the esophagus that do not contain a lumen, as this tissue will be interposed and preclude successful mating of the magnets. Good preparation of the pouches at the time of anastomosis is important.
